# Bilateral tubal ectopic pregnancy following intra uterine insemination (IUI): A case report

**Published:** 2014-02

**Authors:** Mehri Jamilian

**Affiliations:** *Department of Obstetrics and Gynecology, Arak University of Medical Sciences, Arak, Iran.*

**Keywords:** *Ectopic pregnancy*, *Bilateral*, *Intra uterine insemination*

## Abstract

**Background:** The incidence of ectopic pregnancy varies between 1.5-2% of all pregnancies. Bilateral tubal ectopic pregnancy is rare. It may occur in 1 per 200 000 pregnancies.

**Case:** This is a case report of 25 year-old woman who underwent intra uterine insemination (IUI), because of tubal factor infertility (unilateral). On the 30^th^ day after the IUI she complained from pelvic pain and vaginal bleeding. Pelvic ultrasound showed bilateral tubal ectopic pregnancy with fluid in Douglas Pouch and empty uterine cavity. Exploratory laparotomy, left salpingectomy, and right salpingostomy were performed on the same day of admission.

**Conclusion:** The above case suggests that cases presenting with infertility and ectopic pregnancy should be followed very closely with β-hCG and or Trans Vaginal Sonography (TVS) to exclude double ectopic. So, as in this case, early diagnosis is essential for prevention of maternal morbidity and mortality.

## Introduction

The incidence of ectopic pregnancy varies between 1.5-2% of all pregnancies. More than 90% of them occur in the fallopian tubes (1). According to the majority of reports the frequency of ectopic pregnancy has grown in the last 30 years (2). Bilateral tubal ectopic pregnancy is rare and may occur in 1 per 200,000 pregnancies (3, 4). Bilateral simultaneous tubal pregnancy, the rarest form of ectopic pregnancy has also been described following assisted reproduction (5, 6). Since 1918, more than 200 cases of simultaneous bilateral ectopic pregnancies have been reported (6). The last review of the literature on this subject was published by De Los Rios in 2007. Somewhat more than half of those cases were the result of Assisted Reproductive Technique (ART), including ovulation induction, intrauterine insemination, in vitro fertilization and embryo transfer (IVF-ET), transfer of gametes to the fallopian tubes, and intracytoplasmic sperm injections (ICSI) (7-10). 

Complication of ectopic pregnancy remain the leading cause of first-trimester maternal deaths. While most practitioners are familiar with the typical presentation of ectopic pregnancy and manage these cases well, unusual cases may go undiagnosed, and the consequences can be devastating. We report an unusual case of bilateral tubal ectopic pregnancy that occurred in a patient who underwent Intra Uterine Insemination (IUI). This article presents some information that should be useful for the clinician who confronts this rare entity. 

## Case report

A 25-year-old primigravida woman was admitted at the Arak Taleghani Hospital on June 2, 2012 with a history of six weeks amenorrhoea, intermittent vaginal bleeding and mild abdominal pain of two weeks duration, and a positive pregnancy test (five days before admission β-hCG=596, three days before admission β-hCG=2177, and the day of admission β-hCG=3605). There wasn’t history of contraception or previous abdominopelvic surgery. For three years she was taking treatment for infertility. She had undergone IUI, 30 days before admission. She had been married for eight years. 

General examination revealed maternal tachycardia (pulse 110 per min), hypotension (systolic/diastolic blood pressure 90/60 mmHg), and pallor. Her abdomen was tender on palpation with positive rebound and guarding. On pelvic examination, there was mild spotting, the cervicx was closed and the cervical motion was tender. The uterus had normal size. There was fullness in all the fornices with tenderness, and the both adnex were difficult to palpate. Her blood sample was sent for complete blood count (CBC), blood group and Rhesue factor (Rh factor), serum beta subunit of human chorionic gonadotrophin (β-hCG), and renal functions as per protocol.

Hematological examination showed: white cell count 8×10^9^ cells/L, hemoglobin 8.5 g/dl, and hematocrit 25%. Pelvic ultrasound examination showed empty uterus, homogenous texture, mild thickened endometrium, both adnexal mass in both side, and a moderate amount of fluid collection in the Douglas Pouch. A diagnosis of pregnancy of unknown location was made because of lack of a correlation between ultrasound findings and β-hCG levels and because of some clinical signs. She needed emergency intervention. She was taken to operation room for an emergency laparotomy. There was a ruptured ectopic pregnancy with active bleeding on the left side and haemoperitoneum of approximately 800 ml. The right tube showed an intact ectopic pregnancy 2×2.5 cm in the ampullary region with an organized haematoma at the same side. In exploratory laparatomy, we performed left salpingectomy and right salpingostomy. About 300 ml of clots were removed from pelvic cavity by suction. 

The patient received 2 units of packed cell iso group. Patient was stable post operation. Postoperative follow-up was careful and the patient was discharged on the 5^th^ day post-operation. Two weeks after surgery the beta subunit of hCG was zero. Histopathological findings of the specimens, excised the left ruptured fallopian tube and content of right fallopian tube. Decidua and chorionic villi were seen in both sides, with tubal tissue in left side that confirmed bilateral ectopic pregnancy. Informed written consent was obtained from the patient for publication of the report.

**Figure 1:**
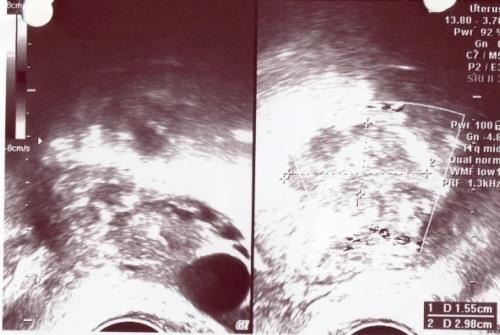
Empty uterus and both adnexal mass in both side

**Figure 2:**
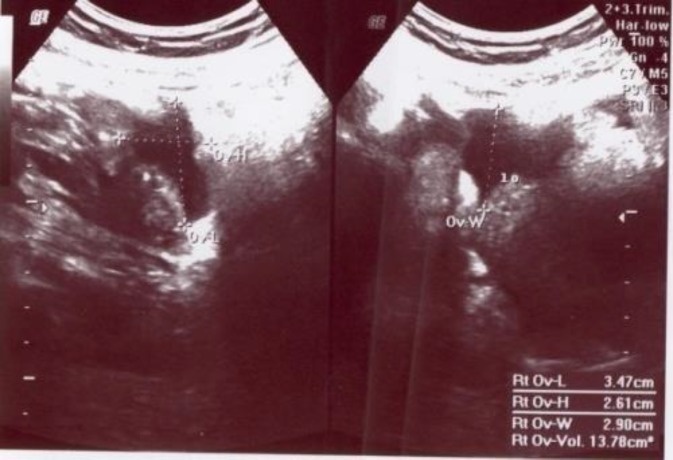
Moderate amount of fluid collection

## Discussion

Bilateral simultaneous tubal pregnancy is an extremely rare form of ectopic pregnancy whose incidence has estimated to be one in 1500 ectopic pregnancies following IUI, therefore preoperative diagnosis is uncommon. The frequency of bilateral ectopic pregnancy is about 1 per 200,000 pregnancies (3, 4). It may occur in 1 per 725-1580 ectopic pregnancies (1, 6). In the past 20 years a 3-fold increase in the incidence has been observed (11). Heterotopic as well as bilateral tubal ectopic pregnancies are seen after the introduction of assisted reproductive technology (12-16). The occurrence of spontaneous bilateral ectopic pregnancy is extremely rare (17-19). 

We reported a very rare case of bilateral simultaneous tubal pregnancy. Pathological findings showed bilateral tubal pregnancy with the presence of products of conception in both fallopian tubes at the time of surgery (20, 21). Ultrasonography of our case failed to make such a diagnosis and this is in agreement with other reports, i.e the use of ultrasound is not necessary to make a diagnosis in bilateral ectopic pregnancy (19, 22). Therefore, early diagnosis of ectopic pregnancy seems to be an important challenge facing emergency physicians. Our findings in this case are similar to those of Kansaria, Chauhan and Mayadeo, who reported a bilateral ruptured ectopic pregnancy (23). 

Surgical management to preserve one of the tubes was performed by salpingostomy in one side and salpingectomy in the other side. We preserve right tube by linear salpingostomy, similar to the above case. Salpingectomy was performed for the left ruptured tube. Steptoe performed the first IVF for a patient with history of tubal factor infertility that was ectopic pregnancy (24). Some cases of bilateral ectopic pregnancy have been reported from 1997 (1, 25-27). The diagnosis of ectopic pregnancy should always be considered in patients undergoing ART, because of its increased incidence compared to natural conception. Although the incidence of a bilateral ectopic pregnancy is rare, both adnex should be examined when diagnosis of an ectopic pregnancy is made (6). 

Treatment of secondary bilateral ectopic pregnancy should not leave any room doubt for the clinician about performing bilateral salpingectomy, especially when such techniques of assisted reproduction as IVF or IUI are used, because, when such a diagnosis is confirmed, bilateral salpingectomy would not only be the treatment of choice but, according to some authors, a condition for the realization of these techniques (28). If the assisted reproduction procedure after treating the bilateral ectopic is going to require the presence of the tubes (i.e., induction of ovulation, intrauterine insemination) and their condition allows it, bilateral salpingostomy could be attempted. 

If, by all means and because of other indications, the patients require the use of other techniques, such as ICSI or IVF-ET, then a good choice is the practice of bilateral salpingectomy. Postoperative results are not well known yet, nor are the reproductive prognoses of any intervention made for the treatment of secondary bilateral ectopic pregnancy (28, 29). The above case suggests that cases presenting with subfertility and ectopic pregnancy should be followed very closely with follow-up tests (β-hCG and TVS) to exclude double ectopic pregnancies. Careful attention reduces the morbidity and mortality of the patient. 

A serial measurement of serum β-hCG is necessary to rule out the risk of persistent trophoblastic. Since the women’s fertility may be affected, we would pay attention to carefully examining of both adnex at the time of exploratory laparotomy undertaken for suspicion of ectopic pregnancy (21, 29). As in this case, early diagnosis is essential for prevention of maternal morbidity and mortality.
